# Laboratory test results and computed tomography findings in health care professionals with COVID-19

**DOI:** 10.1590/0100-3984.2025.0036-en

**Published:** 2026-01-02

**Authors:** Danilo Alves de Araujo, Igor Duarte Pinto Paciello, Samuel Herdy Figueira, Victor Teixeira Ramos Lopes, João Pedro Coelho de Oliveira Barros, Lucas Vazquez Barreira Ranzeiro de Bragança, Matheus Rodrigues Miranda, Alair Augusto Sarmet Moreira Damas dos Santos

**Affiliations:** 1 Faculdade de Medicina da Universidade Federal Fluminense (UFF), Niterói, RJ, Brazil

**Keywords:** COVID-19, Health personnel, Tomography, X-ray computed, Thorax., COVID-19, Profissionais de saúde, Tomografia computadorizada, Tórax.

## Abstract

**Objective:**

To assess the intensity, characteristics, and distribution of computed tomography (CT) findings of pulmonary involvement, as well as to evaluate laboratory test results, in health care professionals who were exposed to severe acute respiratory syndrome coronavirus 2 (SARS-CoV-2).

**Materials and Methods:**

This was a retrospective, cross-sectional, observational study based on the analysis of laboratory test results and chest CT images of health care workers with confirmed coronavirus disease 2019 (COVID-19). Data for the period from March 2020 to December 2022 were collected from two hospitals in Brazil.

**Results:**

We identified 1,091 health care professionals in whom a RT-PCR was positive for SARS-CoV-2. However, only 38 of those individuals underwent chest CT. Of the 38 individuals evaluated, 89.5% were treated at one of the hospitals and 57.9% were male. The mean age was 55.6 years. The most common finding (in 100% of the cases) was ground-glass opacity, followed by septal thickening (in 31.6%) and consolidation (in 23.7%). Pulmonary involvement was multifocal in 76.3% and predominantly subpleural in 71.0%. The extent of the involvement was classified as mild in 24% of the cases, moderate in 47%, and severe in 29%. The most commonly affected lung region (in 60.7% of cases) was the lower lobes, particularly the right posterior basal segmental bronchus (segment B10).

**Conclusion:**

For evaluating lung involvement, CT was essential, aiding in postinfection monitoring and in the early management of complications. Among the health care professionals evaluated, moderate involvement predominated.

## Abstract (pt)

**Objetivo:**

Avaliar a intensidade, as características e a distribuição dos achados tomográficos do acometimento pulmonar, bem como os dados laboratoriais, em profissionais de saúde expostos ao SARS-CoV-2.

**Materiais e Métodos:**

Estudo transversal, observacional, retrospectivo, baseado na análise de exames laboratoriais e de imagens de tomografia computadorizada de tórax de profissionais de saúde com COVID-19 confirmada, atendidos em dois hospitais brasileiros entre março de 2020 e dezembro de 2022.

**Resultados:**

Foram identificados 1.091 profissionais de saúde com RT-PCR positivo para SARS-CoV-2, no entanto, somente 38 deles realizaram tomografia de tórax. Desses 38 casos, 89,5% eram de um dos hospitais, 57,9% do sexo masculino, com média de idade de 55,6 anos. O padrão em vidro fosco foi o achado tomográfico mais frequente (100%), seguido de espessamento septal (31,6%) e consolidação (23,7%). O acometimento pulmonar mostrou-se multifocal em 76,3% dos exames, predominante em regiões subpleurais (71%). Quanto à extensão das lesões, 24% dos casos foram classificados como leve, 47% como moderada e 29% como grave. O lobo inferior direito foi o mais afetado (60,7%), com o maior envolvimento no segmento basal posterior do pulmão direito (segmento B10).

**Conclusão:**

A tomografia computadorizada mostrou-se essencial para avaliar o acometimento pulmonar, auxiliando no monitoramento pós-infecção e no manejo precoce de complicações. Nos profissionais de saúde avaliados, predominou o acometimento moderado.

## INTRODUCTION

On December 31, 2019, the Wuhan Municipal Health Commission issued a statement on cases of “viral pneumonia” in the city, marking the beginning of the coronavirus disease 2019 (COVID-19) pandemic, which has since transformed lives and health care systems worldwide^(1)^. The disease, caused by infection with severe acute respiratory syndrome coronavirus 2 (SARS-CoV-2), has a variable clinical spectrum, ranging from asymptomatic cases to severe cases with persistent respiratory complications, such as pulmonary fibrosis, known as long COVID^(2)^. Although the incubation period for SARS-CoV-2 is typically four to five days, symptoms can appear up to 14 days after exposure^(3,4)^.

The COVID-19 pandemic has affected millions of people globally, with significant impacts on public health and the economy^(5)^. According to the World Health Organization, the disease can manifest with symptoms such as fever, dry cough, dyspnea, anosmia, and dysgeusia, as well as gastrointestinal symptoms in some cases^(6,7)^. Risk factors for severe complications include advanced age, obesity, hypertension, diabetes mellitus, and pre-existing comorbidities^(8)^. Biochemical abnormalities such as lymphopenia, elevated lactate dehydrogenase (LDH), and elevated D-dimer are associated with a worse prognosis^(9)^. Infection with SARS-CoV-2 is confirmed by reverse transcription-polymerase chain reaction (RT-PCR), which detects viral RNA in nasopharyngeal swab samples, although its sensitivity depends on the collection technique and the viral load at the time of testing^(10)^.

Health care professionals, especially those on the front lines, faced a high risk of transmission, with infection rates reaching over 40% in one study conducted at the height of the pandemic^(11)^. Despite the use of personal protective equipment and vaccination, which significantly reduced infection and hospitalization rates^(12,13)^, many health care professionals developed severe or chronic symptoms, which highlights the dire need for clinical and radiological monitoring that is more individualized^(14)^.

Chest computed tomography (CT) has emerged as a crucial tool in the diagnosis and monitoring of COVID-19, especially in severe cases or when pulmonary complications are suspected^(15,16)^. Findings such as ground-glass opacities, consolidations, and a subpleural distribution are frequently observed, particularly in the lower lung lobes^(17)^. On chest CT, abnormalities suggestive of viral pneumonia have been identified early, even before the development of symptoms and the detection of viral RNA^(15)^. These radiological findings have been essential for early diagnosis, as well as for the identification of cases that are more likely to have an unfavorable evolution^(12,13,18)^.

The aim of this study was to investigate CT and biochemical characteristics in health care professionals exposed to SARS-CoV-2 in Brazil, comparing the findings with those of international studies. The analysis aims to bridge a gap in the national literature, providing insights into the impact of COVID-19 on this highly exposed group, as well as contributing to clinical management and case monitoring. The hypothesis was that health care professionals infected with SARS-CoV-2 present specific CT and biochemical alterations, such as ground-glass opacities, lymphopenia, and elevated levels of inflammatory markers (C-reactive protein, LDH, and D-dimer), which are associated with a higher risk of persistent pulmonary complications, especially in individuals with severe forms of the disease.

## MATERIALS AND METHODS

This was a retrospective, cross-sectional, observational study, conducted at two hospitals in the city of Niterói, in the state of Rio de Janeiro, Brazil, with the aim of investigating the CT and biochemical characteristics of health care professionals with COVID-19. This work was approved by the local and national research ethics committees (Reference no. 34014720.6.0000.5289).

### Study population

All health care professionals treated for SARS-CoV-2 infection, confirmed by RT-PCR, between March 2020 and December 2022 at the participating institutions were considered eligible for inclusion in the study. Only those who developed COVID-19 and underwent chest CT during the acute phase of the disease were included in the imaging analysis. Cases were excluded if there were no available imaging examination results.

### Data collection

Chest CT scans were acquired in multidetector scanners, with the individual in the supine position, without contrast and with 5-mm slices. Most CT scans were analyzed by two experienced radiologists, working independently. Disagreements were resolved by consensus, as well as by conducting a systematic review of the imaging examinations and searching for the most common patterns.

The following laboratory tests were performed during the acute phase of the disease: complete blood count (with emphasis on total leukocyte count and absolute lymphocyte count); C-reactive protein; LDH; aspartate aminotransferase (AST); alanine aminotransferase (ALT); D-dimer; and serum creatinine. The reference values adopted were those used by the clinical laboratories of the institutions, as shown in Table 1. We considered the initial values from these tests, performed at hospital admission or within one week after the diagnosis, as available in the medical records, as the baseline values.

**Table 1:** Laboratory test results.

Parameter	n	Reference range	Mean ± standard deviation	High n (%)	Low n (%)	Normal n (%)
Leukocyte count (x 10^9^/L)	32	4.0-10.0	6.36 ± 2.12	3 (9.38)	6 (18.75)	23 (71.87)
Lymphocyte count (x 10^9^/L)	32	1.1-3.2	1.29 ± 0.67	1 (3.12)	17 (53.12)	14 (43.75)
C-reactive protein (mg/L)	30	0-3.0	4.90 ± 5.84	13 (44.33)	No data	17 (56.66)
LDH (IU/L)	30	114-240	235 ± 72.79	16 (53.33)	0 (0.0)	14 (46.66)
AST (IU/L)	13	0-45	40.29 ± 20.06	5 (38.46)	No data	8 (61.53)
ALT (IU/L)	15	9-0	45.00 ± 26.54	8 (53.33)	0 (0.0)	7 (46.66)
D-dimer (pg/mL)	21	0-0.5	0.83 ± 0.59	14 (66.67)	No data	7 (33.33)
Creatinine (mg/L)	26	0.5-1.3	1.21 ± 0.33	9 (34.61)	0 (0.0)	15 (57.69)

### Quantification of lung involvement

Lung involvement was quantified with a scoring scheme based on dividing the lungs into right and left segments, corresponding to the anatomical segments. The absence of lung involvement received a score of zero, and each affected lung segment received one point. The total score ranged from 0 to 20, characterizing the lung involvement as mild (1-5 points), moderate (6-10 points), or severe (> 10 points).

### Statistical analysis

Descriptive analysis of the data was performed. Continuous variables are presented as mean and standard deviation, whereas categorical variables are presented as absolute frequencies and percentages.

## RESULTS

Of the 1,091 health care professionals who tested positive for infection with SARS-CoV-2 on RT-PCR during the study period, 38 (3.5%) underwent chest CT and composed the study sample. The mean age was 55.61 ± 14.14 years (range, 32-79 years). Of the 38 participants, 57.9% were men and 42.1% were women. All of the participants were residents of the state of Rio de Janeiro and were employed at one of the two facilities investigated (hereafter referred to as hospitals A and B), four (10.5%) at hospital A and 34 (89.5%) at hospital B.

Table 1 shows the data from laboratory analyses relating to leukocyte, lymphocyte, C-reactive protein, LDH, AST, ALT, creatinine, and D-dimer counts in the patients. Six patients did not undergo any laboratory tests.

The patients presented some laboratory alterations associated with a worse prognosis, such as leukocytosis (in 9.4%), leukopenia (in 18.8%), lymphopenia (in 53.1%), elevated C-reactive protein (in 44.3%), elevated LDH (in 53.3%), elevated D-dimer (in 67.7%), elevated AST (in 38.5%), elevated ALT (in 53.3%), and elevated creatinine (in 34.6%).

The radiological patterns most commonly observed on the chest CT scans were ground-glass opacity (in 100%), septal thickening (in 31.6%), consolidation (in 23.7%), parenchymal band (in 21.0%), air bronchogram (in 18.4%), and pleural effusion (in 10.5%).

**Figure 1:**
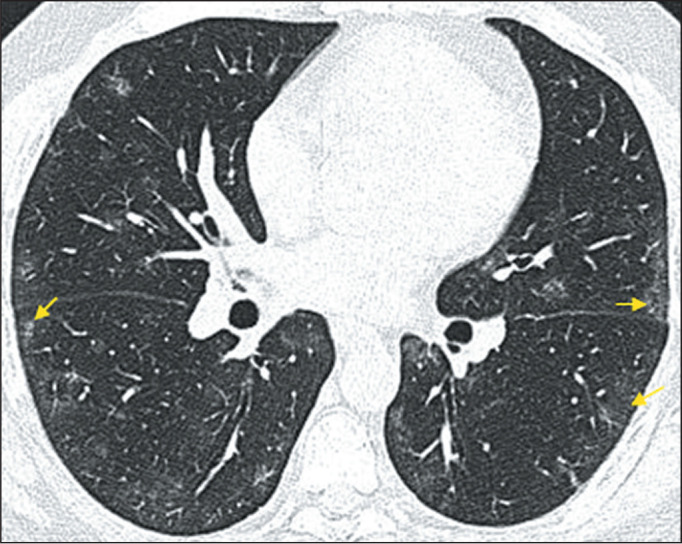
CT scan of a 47-year-old male patient, showing discrete ground-glass opacities (arrows), with a multifocal and predominantly subpleural distribution.

**Figure 2:**
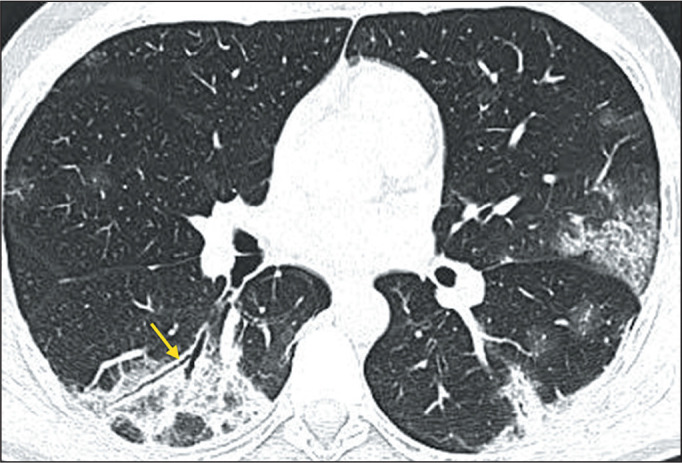
CT scan of a 39-year-old female patient, showing ground-glass opacities and a crazy-paving pattern, together with consolidation and air bronchograms in segment B6 (arrow).

**Figure 3:**
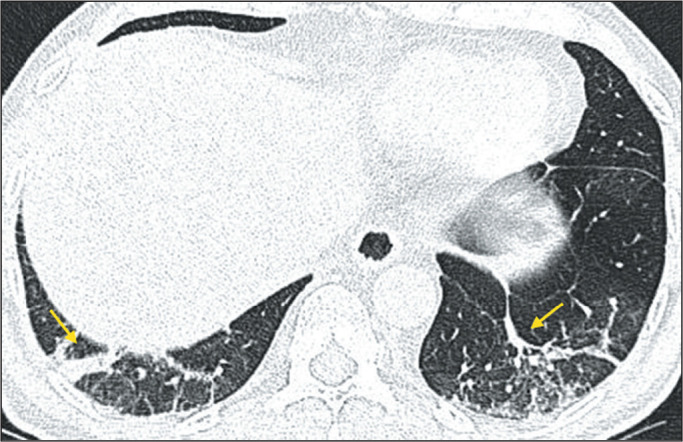
CT scan of a 54-year-old male patient, in a later disease stage, showing parenchymal bands (arrows).

Ground-glass opacity (Figure 1) is characterized by increased lung density that does not obscure the internal vascular structures and should be differentiated from consolidation, in which the vessels are not visible. When accompanied by thickening of the interlobular septa, it forms what is known as the crazypaving pattern, which was not observed in our study sample. An air bronchogram (Figure 2) is defined as visible aerated bronchi within areas of consolidation or atelectasis. The interlobular septa, which delimit the secondary pulmonary lobule, are composed of connective tissue, pulmonary veins, and lymphatic vessels; the septa can present smooth, irregular, or nodular thickening in conditions such as edema, inflammation, fibrosis, and neoplasia. A parenchymal band (Figure 3) is an elongated linear opacity, commonly peripheral and accompanied by fibrosis or interstitial thickening, frequently in contact with the pleura, which can present thickening and retraction^(19)^.

Most (76.3%) of the examinations showed multifocal involvement, with diffuse involvement being seen in the remaining nine examinations (23.7%). The distribution of lung involvement, in terms of location, was subpleural in 71% of the cases (Figure 1), being peribronchial or diffuse in 29%. As detailed in Table 2, the extent of lung involvement was classified as follows: mild (up to 25%), moderate (25-50%), or severe (over 50%).

**Table 2:** Classification of the extent of lung involvement.

Category	(N = 38)
Mild, n (%)	9 (23.7)
Moderate, n (%)	18 (47.4)
Severe, n (%)	11 (28.9)

The scoring for the affected lung segments ranged from 1 to 19 points. When comparing the upper and lower lung lobes, we found that the lower lobes accounted for 60.7% of the points scored, compared with 39.3% for the upper lobes. The point distribution between the right and left lobes was more comparable (54.7% vs. 45.3%). The right lower lobe accounted for 33.8% of the total score, with the posterior basal segmental bronchus (B10) being the most commonly affected, followed by the lateral basal segmental bronchus (B9) and the superior segmental bronchus (B6).

Opacity scores were analyzed according to the covariates associated with greater severity, such as consolidation, lymphopenia, elevated C-reactive protein, and elevated LDH. The correlations with the extent of lung involvement were as follows: consolidation (*p* = 0.013); lymphopenia (*p* = 0.054); C-reactive protein (*p* = 0.062); and LDH (*p* = 0.226). Therefore, only consolidation presented statistical significance for the extent of lung involvement. Tables 3, 4, 5, and 6 show comparisons between the present study and two previous studies-Liu et al.^(20)^ and Xiong et al.^(21)^-in terms of the laboratory test results, the CT findings, the dissemination of lung involvement, and the location of the lung involvement, respectively.

**Table 3:** Biochemical alterations.

Study	Leukopenia	Lymphocytopenia	C-reactive protein ↑	LDH ↑	AST ↑	ALT ↑	D-dimer ↑
Present study	18.75%	53,12%	44.33%	53.33%	38.46%	53.33%	66.67%
Liu et al.^(20)^	18%	35%	48%	13%	23%	13%	17%
Xiong et al.^(21)^	39.5%	48.8%	51.2%	44.2%	No data	No data	No data

**Table 4:** Most common patterns of lung involvement.

Study	Ground-glass opacity	Septal thickening	Consolidation	Parenchymal bands	Air bronchogram	Pleural effusion
Present study	100%	31.6%	23.7%	21.0%	18.4%	10.5%
Liu et al.^(20)^	54-81%	No data	18%	3-32%,	No data	3-5%
Xiong et al.^(21)^	No data	32.5%	37.2%	No data	18.6%	3%

**Table 5:** Distribution of pulmonary involvement according to the pattern of dissemination (multifocal or diffuse).

Study	Multifocal	Diffuse
Present study	76.3%	23.7%
Liu et al.^(20)^	67-88%	12-33%
Xiong et al.^(21)^	60.50%	39.50%

**Table 6:** Distribution of pulmonary involvement according to the location (subpleural, peribronchial, or mixed).

Study	Subpleural	Peribronchial	Mixed
Present study	71.0%	15.8%	13.2%
Liu et al.^(20)^	76-78%	No data	No data
Xiong et al.^(21)^	41.9%	25.6%	30.2%

## DISCUSSION

In this study, we have demonstrated that, among health care professionals with COVID-19 who underwent chest CT, the pattern of ground-glass opacities with peripheral, multifocal distribution predominated, with moderate lung involvement in most. Laboratory findings included lymphopenia and elevated levels of inflammatory markers (C-reactive protein, LDH, and D-dimer).

The studies conducted by Liu et al.^(20)^ and Xiong et al.^(21)^ also used laboratory testing to highlight altered parameters in health care professionals with COVID-19. The comparison of the laboratory data revealed that the proportion of individuals with leukopenia in the present study was similar to that reported by Liu et al.^(20)^, whereas it was only half as high as that reported by Xiong et al.^(21)^. The prevalence of lymphocytopenia was highest in the present study, followed by that of Xiong et al.^(21)^. Elevated C-reactive protein was observed in approximately half of the participants in all three studies. Elevated LDH was identified in approximately half of the cases in the present study and in that of Xiong et al.^(21)^, whereas a lower proportion was observed in the study of Liu et al.^(20)^. The proportions of participants with elevated AST, ALT, and D-dimer levels were higher in the present study than in the study conducted by Liu et al.^(20)^. Those parameters were either not evaluated or not provided as proportions in the study conducted by Xiong et al.^(21)^. The higher proportions found in the present study might be explained by the fact that, in our study sample, the examinations were performed mainly in patients in whom the clinical suspicion was high before the test was performed. Other studies have also reported data similar to those observed in the present study, including those related to lymphocytopenia, as well as those related to elevated levels of LDH, C-reactive protein, and D-dimer^(22,23)^.

Pulmonary involvement by COVID-19 is predominantly characterized by ground-glass opacity, and this feature was consistently observed in the present study, as well as in the studies conducted by Liu et al.^(20)^ and Xiong et al.^(21)^. It is noteworthy that Liu et al.^(20)^ identified ground-glass opacity in all phases of acute infection in at least 50% of the cases analyzed. In the study conducted by Xiong et al.^(21)^ and in the present study, septal thickening and air bronchogram were found in similar proportions. Pulmonary consolidation, associated with disease progression in the context of SARS-CoV-2 infection, was observed in the highest proportion in the study conducted by Xiong et al.^(21)^, followed by the present study, and finally by the study conducted by Liu et al.^(20)^. Parenchymal bands, often associated with chronic changes, were observed in almost one-third of the later-stage cases in the study conducted by Liu et al.^(20)^. Bronchiectasis and a tree-in-bud pattern were not observed in any of the three studies compared.

The terms septal thickening and air bronchogram were not adopted in the study conducted by Liu et al.^(20)^, whereas parenchymal bands were not mentioned in the study conducted by Xiong et al.^(21)^, which also did not include the proportion of examinations showing ground-glass opacity. The reversed halo sign was not reported in any of the three studies.

Multifocal distribution was the most common pattern seen on CT, with a prevalence exceeding 60% in all three studies and that prevalence being highest in the study conducted by Liu et al.^(20)^, whereas the proportion of CT scans showing a diffuse pattern of distribution was highest in the Xiong et al.^(21)^ study. Subpleural involvement constituted the main location observed in all three studies, notably in the present study and in that conducted by Liu et al.^(20)^. In the Xiong et al.^(21)^ study, the mixed pattern was more common than was the peribronchial pattern, which was not observed in the present study. Regarding the extent of lung involvement by segment, Liu et al.^(20)^ identified the B6 segment as the most affected, followed by the B10 segment and the B9 segment. Similarly, Xiong et al.^(21)^ found that that most of the alterations were in the lower lobes. Liu et al.^(20)^ did not describe the proportional distribution of peribronchial or mixed involvement.

During the COVID-19 pandemic, chest CT played a crucial role in the evaluation and monitoring of patients with suspected or confirmed COVID-19. The initial shortage of diagnostic tests led to chest CT becoming one of the primary methods for confirming suspected infection with SARS-CoV-2^(16)^. Health care professionals on the front lines may have undergone chest CT to evaluate respiratory symptoms or suspected infection more frequently than the general population, given their greater access to the examination. The retrospective study conducted by Xiong et al.^(21)^ demonstrated that the alterations seen on initial chest CT scans were less pronounced among health care professionals, which could be attributed to the use of personal protective equipment and likely earlier access to imaging^(21)^. When we began this study, we believed that CT findings in health care professionals would be milder than those reported for the general population. However, we found that there were cases of moderate and severe disease within our study sample. We found that factors such as advanced age, pre-existing comorbidities, and prolonged exposure to infected individuals increased the risk of the severe forms of COVID-19 among health care professionals, corroborating findings from international studies^(24)^.

Our imaging findings regarding the extent and pattern of lung parenchymal involvement, such as the multifocal and subpleural areas in the lower lobes with ground-glass opacity, are consistent with those of previous studies^(11,20,21)^, which also highlight the persistence of CT changes even after clinical recovery^(14,24)^.

The Fleischner Society^(25)^ and the Brazilian College of Radiology and Diagnostic Imaging^(26)^ have published consensus statements on the use of chest imaging in the context of COVID-19. In asymptomatic patients or those with mild respiratory symptoms, chest imaging is not indicated as screening. It is indicated only in patients with moderate to severe respiratory symptoms, those > 65 years of age, and those with comorbidities such as cardiovascular disease, diabetes, chronic disease, respiratory disease, hypertension, and immunocompromised status.

Our results underscore the role of CT as a useful tool in the management of COVID-19 in health care professionals, allowing for a rapid assessment of the extent of pneumonia. In particular, our finding that the majority of health care professionals presented with moderate involvement could guide the follow-up of those with severe involvement, who may require prolonged leave and monitoring by a pulmonologist because of the increased risk of fibrosis. The high proportion of professionals with an elevated D-dimer level suggests the need for attention to thromboembolic risk even in this group.

Our study has some limitations that should be considered when the results are interpreted. First, it was a retrospective study, which means that data were collected from medical records and examinations performed previously, without the possibility of controlling the data collection or standardizing the procedures. In addition, we did not monitor the long-term outcomes of the cases evaluated. We do not know, for example, how many individuals developed pulmonary fibrosis or other late outcomes, given our cross-sectional design. Future studies could follow health care professionals during the post-COVID phase to assess sequelae, particularly in those with severe initial involvement. Furthermore, participant selection was based on convenience, including only health care professionals who underwent chest CT scans, as requested by their attending physician. Therefore, the participants evaluated may not represent all health care professionals infected with SARS-CoV-2, but rather those who presented symptoms or clinical conditions that justified the examination. As a consequence, the sample may be biased toward cases that were symptomatic or more severe, limiting the generalizability of the results to health care professionals with asymptomatic or mild forms of the disease. Another important limitation is that no additional tests were performed exclusively for research purposes, which could have allowed a more comprehensive and standardized assessment of the CT findings and laboratory test results. Reliance on tests requested for clinical reasons may have resulted in an underestimation or overestimation of some alterations, depending on the clinical indication for chest CT. We did not have access to detailed data on previous comorbidities, medication use, vaccination status, or virus variant, factors that can influence the clinical and radiological presentation. Those uncontrolled variables are potential confounders.

The COVID-19 pandemic created a scenario of high demand for diagnostic and therapeutic resources, which may have influenced the decision to request imaging examinations only for cases in which there was a high suspicion of pulmonary complications. That could explain the discrepancy between the total number of health care professionals with a positive RT-PCR and those who underwent chest CT, who accounted for only 3.5% of the total sample. Many health care professionals may have undergone examinations at other institutions or may not have had access to chest CT because of the overloaded health care system during the pandemic.

Another point to consider is the scarcity of studies on CT findings in health care professionals infected with SARS-CoV-2. Most available studies focus on the general population or specific risk groups, such as the elderly or patients with comorbidities. That makes it difficult to draw direct comparisons between the findings of the present study and those of other studies, especially regarding the specific clinical and radiological characteristics of health care professionals.

Despite the limitations outlined above, our findings fill the gap identified in the national literature on COVID-19 in health care professionals, providing an initial characterization of this group in the context of two hospitals in Brazil.

Our findings highlight the importance of detailed clinical and radiological follow-up for health care professionals who develop severe forms of COVID-19. Post-COVID-19 follow-up is crucial for identifying and managing persistent pulmonary complications, such as fibrosis and bronchiectasis, which can significantly impact the quality of life of health care professionals^(14)^. The presence of ground-glass opacities, consolidations, and subpleural distribution on chest CT, together with biochemical alterations such as lymphopenia, elevated LDH, and elevated C-reactive protein, suggest that these individuals may be at higher risk for persistent pulmonary complications, such as pulmonary fibrosis. Therefore, the implementation of postinfection monitoring protocols, including periodic radiological evaluation and laboratory tests, may be crucial for the early detection and appropriate management of long-term sequelae. In addition, early identification of health care professionals at higher risk of developing severe forms of the disease can guide prevention and intervention strategies, such as prioritizing booster vaccination and the early use of antiviral therapies, which could reduce morbidity and mortality in this highly exposed population. Future studies should investigate not only the physical sequelae but also the psychosocial impact that COVID-19 has on health care professionals, as well as strategies to mitigate such effects^(19)^.

## CONCLUSION

Chest CT has proven to be a valuable tool in the evaluation of health care professionals with COVID-19, demonstrating typical patterns of viral pneumonia (multifocal subpleural ground-glass opacities) and quantifying the extent of lung involvement, which was moderate in most of the cases evaluated here. Laboratory tests in those professionals corroborated the systemic inflammatory activity of the disease, often revealing lymphopenia and elevated levels of markers such as C-reactive protein, LDH, and D-dimer. Taken together, these findings suggest that an integrated imaging-laboratory testing approach may aid in postinfection monitoring and early management of potential complications, especially in the more severe cases.

## Data Availability

Data set related to this study are published in the body of this article.
